# Integrating phenotype and gene expression data for predicting gene function

**DOI:** 10.1186/1471-2105-10-S11-S20

**Published:** 2009-10-08

**Authors:** Brandon M Malone, Andy D Perkins, Susan M Bridges

**Affiliations:** 1 Department of Computer Science and Engineering, Box 9637, Mississippi State University, Mississippi State, MS, 39762 USA; 2 Institute for Digital Biology, Mississippi State University, Mississippi State, MS, 39762 USA

## Abstract

**Background:**

This paper presents a framework for integrating disparate data sets to predict gene function. The algorithm constructs a graph, called an integrated similarity graph, by computing similarities based upon both gene expression and textual phenotype data. This integrated graph is then used to make predictions about whether individual genes should be assigned a particular annotation from the Gene Ontology.

**Results:**

A combined graph was generated from publicly-available gene expression data and phenotypic information from *Saccharomyces cerevisiae*. This graph was used to assign annotations to genes, as were graphs constructed from gene expression data and textual phenotype information alone. While the F-measure appeared similar for all three methods, annotations based upon the integrated similarity graph exhibited a better overall precision than gene expression or phenotype information alone can generate. The integrated approach was also able to assign almost as many annotations as the gene expression method alone, and generated significantly more total and correct assignments than the phenotype information could provide.

**Conclusion:**

These results suggest that augmenting standard gene expression data sets with publicly-available textual phenotype data can help generate more precise functional annotation predictions while mitigating the weaknesses of a standard textual phenotype approach.

## Background

With the advent the "omics technologies," researchers are faced with the problem of analyzing high throughput datasets. The Gene Ontology (GO) was initiated to provide a controlled vocabulary for describing the cellular location, biological process, and molecular function of gene products and to thus enable extraction of biological meaning from these large datasets [1]. The terms in the GO are organized in a directed acyclic graph where directed edges represent relationships among terms. The primary relationships between terms in the GO are "part_of" and "is_a". Assignment of a GO term to a gene product is called annotation. GO annotation has become a "gold standard" in describing and the function of gene products and in supporting computational methods for analyzing high throughput datasets.

Assigning GO terms to gene products has now become a major bottleneck in the analysis of large datasets and has prompted the development of many computational approaches. The Gene Ontology Annotation (GOA) project [2] employs a pipeline which incorporates both manually curated and electronic approaches to annotate UniProtKB entries with GO terms. The manual assignment of annotations relies on curators searching through literature for evidence that a protein has a particular function. While this process can be slow and expensive, the results are typically very accurate and detailed. The electronic aspect of the pipeline incorporates results from a variety of sources including Swiss-Prot keywords, cross references to InterPro, and orthology mapping from a source species to a target species. Electronic annotation is particularly useful for the assignment of GO terms to the proteins of non-model organisms which likely would not receive manual annotations. Many other computational annotation pipelines for assignment of Gene Ontology terms have been developed. For example, DAVID [3] agglomerates data from many sources, both manually curated and computationally populated, into a single database. CLUGO [4] utilized homology search combined with clustering to assign terms to new sequences. Text mining is also frequently used to computationally predict gene functions with the goal of automating the manual process of annotating gene products from the literature. For example, Daraselia et al. [5] automatically extract functional annotations for mammalian proteins from Medline texts by building regular expression to find relationships between GO terms and proteins. Groth et al. [6-8] use text mining to associate phenotypes with genes by clustering term frequency-inverse document frequency (tf-idf) arrays. Functional predictions are inferred for all genes in a cluster for a particular annotation when at least half of the genes in the cluster had that annotation. In addition to sequence and text data, gene expression data is also often used in predicting functional annotations. For example, Virtual Gene Ontology (VIRGO) [9] constructs functional linkage networks (FLNs) in which nodes in a graph represent genes and edges indicate the Pearson correlation between the expression arrays of genes. Functional annotations are propagated across the network by treating the network as a discrete Hopfield network [10].

We present a new algorithm that combines text mining of phenotypic data with inference based on gene expression patterns to predict whether a particular gene should receive a particular GO annotation based on its similarity to other genes known to have the annotation. We demonstrate the utility of our approach with the well-annotated yeast genome where current annotations are considered the "true annotation." The algorithm will be most useful, however, for annotating gene products of less well studied organisms without large research communities.

## Methods

Our algorithm first computes the similarity of all genes under consideration based on two types of data: phenotype extracted using text mining and gene expression profiles. A complete graph is then constructed where each vertex corresponds to a gene and the weights on edges represent the similarity of a pair of genes. Assignment of a GO annotation is determined for each gene based on the similarities to other genes with this annotation.

### GO annotations

In order for the algorithm to predict functions associated with unlabeled genes, it must have existing labels to use as a training set. This algorithm uses current GO annotations as labels [1]. The notation *annotation(a, g) *indicates that gene *g *has annotation *a*.

### Similarity functions

A similarity graph is used to integrate multiple data sources to predict whether a gene should receive a particular GO annotation. Similarity functions form the basis of the prediction algorithm. A similarity function takes as input a representation of two genes and returns a value between -1 and 1 reflecting the similarity between the two genes, where -1 represents high dissimilarity and 1 indicates high similarity. More specifically, a similarity function is defined for each data set. Thus, integrating *n *data sets requires *n *similarity functions. The functions need not be distinct. So, *f*: *G *× *G *→ [0, 1], where *f *is a similarity function and *G *is the set of genes.

### Gene expression similarity function

The similarity function for the gene expression data between two genes is defined as the Pearson correlation coefficient of the associated expression arrays of the two genes [11]. Each gene expression array will typically represent the expression level of a gene under different conditions or at different time points in a treatment. The assumption is that genes exhibiting similar expression profiles will have similar functions. The Pearson correlation coefficient between two arrays is defined as(1)

where *x*_*i *_is an element from the array *x*,  is the mean of the elements in the array *x*, *y*_*i *_is an element from the array *y*,  is the mean of the elements in the array *y*. Because negatively correlated genes are not informative for assigning GO terms to genes[12], negative correlation values are replaced by 0. Therefore, the similarity of two genes based on expression is defined as(2)

where *v*_*i *_and *v*_*j *_are arrays representing gene expression profiles for genes *g*_*i*_and *g*_*j*_, respectively.

### Textual phenotype similarity functions

Because the textual phenotypes are not easily amenable to computation, some method to transform the text into a usable form is necessary. Term frequency – inverse document frequency (tf-idf) arrays offer one common approach for turning text into arrays [13]. The term frequency (tf) indicates how many times a particular term appears in a particular document; the intuition asserts that terms which appear often in a document more accurately describe that document [13]. As a term appears in more documents, though, that term carries less information. The inverse document frequency (idf) accounts for this phenomena [13]. The idf is the log of the total number of documents divided by the number of documents containing the term. The tf-idf value for a term is the product of the tf and idf. Stop lists are used to remove common words such as articles and prepositions and stemming algorithms reduce alternate tenses and forms of words to a single root form [13]. The tf-idf array for a gene contains the tf-idf values for all possible terms in the complete set of documents (corpus). The assumption is that genes with similar tf-idf arrays will have similar functions. The similarity function for the textual phenotype data is defined as the cosine distance between the tf-idf arrays associated with two genes [13] given by(3)

where *v*_*i *_and *v*_*j *_are tf-idf arrays associated with genes *g*_*i *_and *g*_*j*_, respectively.

### Constructing the similarity graph

A graph is constructed by creating a vertex to represent each gene symbol. The graph is then completely connected and the weight of each edge represents the similarity of the genes corresponding to the vertices. The weight of the edge is calculated in two different ways. The first method uses the sum of the values of all similarity functions between the two genes:(4)

where *w*_*i*, *j *_is the weight of the edge between the vertices representing genes *g*_*i *_and *g*_*j*_, and *f*_*k *_is a similarity function based on one of the data sets. The second method constructs a graph with the same vertices and edges, but instead of using the sum of the similarity functions to find the weights, the max is taken instead. That is:(5)

### Predicting functional annotations

**Figure 1:**
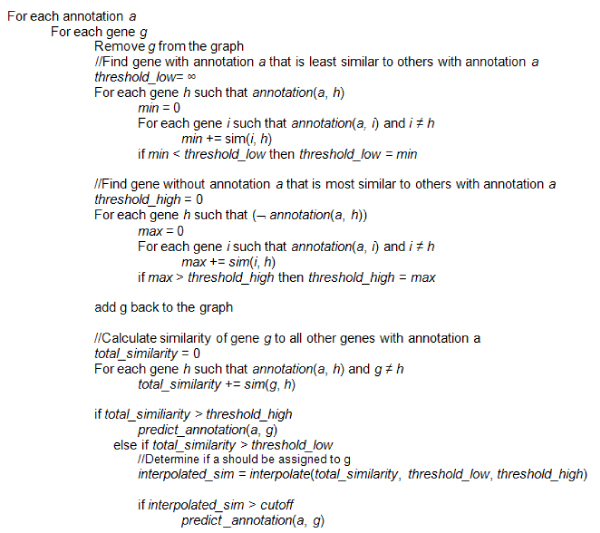
**Pseudocode for annotation prediction algorithm**. The algorithm predicts whether gene *g *should be annotated with annotation *a*. The algorithm consists of four key steps. First, *threshold_low*, which is the lowest similarity of any gene known to have *a *to all other genes known to have *a*, is calculated. Next, *threshold_high*, which is the highest similarity of any gene known to not have *a *to all genes known to have *a*, is calculated. Then, *total_similarity *of *g *to all genes known to have *a *is calculated. Finally, the prediction is made. If *total_similarity *exceeds *threshold_high*, then *g *is always predicted to have annotation *a*. If *total_similarity *is less than *threshold_low*, then *g *is never predicted to have annotation *a*. If *total_similarity *falls between *threshold_low *and *threshold_high*, then it is linearly interpolated between the two thresholds to produces a number between 0 and 1. Specifically, the formula for the linear interpolation is . An predefined *cutoff*, such as 0.5, is then used to predict whether or not to assign the annotation to gene *g*. Thus, if *cutoff *= 0.5 and *interpolated_sim *= 0.6 for gene *g *and annotation *a*, then gene *g *would be predicted to have annotation *a*.

**Figure 2:**
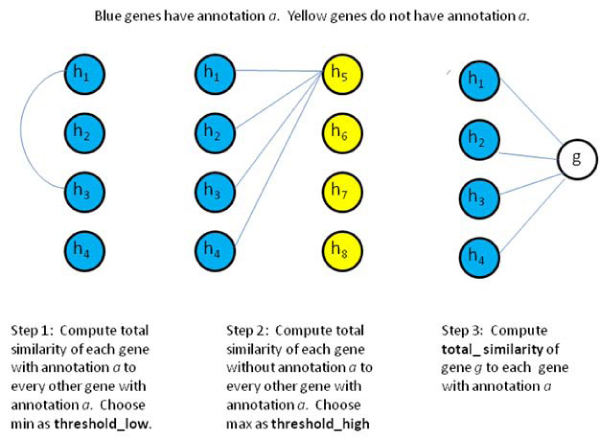
**Computing key similarities for predicting annotations**. Three key similarity calculations are required to determine if annotation *a *should be assigned to gene *g*. Step 1: The lower threshold is the minimum of the total similarity of each gene with annotation *a *to all other genes with annotation a. Step 2: The upper threshold is the maximum of the total similarity of each gene without annotation *a *to every gene with annotation *a*. Step 3: The total similarity of gene *g *to all genes with annotation a is computed and the decision for assigning annotation *a *to gene *g *is made as illustrated in Figure 3.

Prediction of whether a particular gene should receive a particular GO annotation is made using the complete graphs constructed as described above. The nodes in the graph correspond to genes, and the weights of its edges correspond to the similarity between genes. These similarities are derived using the gene expression and phenotype similarity functions described above. Figure 1 gives the pseudocode for the prediction algorithm. The same prediction algorithm can be used regardless of whether the sum or the max was used to calculate edge weights. For each annotation *a*, we consider each gene *g *in turn and determine whether the annotation should be assigned to the gene. For a particular gene and annotation pair (*g*, *a*), we first remove gene *g *from consideration and then compute two thresholds. Figure 2 illustrates the key similarity computations. In step 1, a lower threshold on similarities is computed by finding the gene *h *with annotation *a *with the smallest total similarity to other genes with this annotation.(7)

We consider this a lower bound and will not assign the annotation to gene *g *if it has a total similarity to other genes with annotation *a *lower than this threshold.

As shown in Figure 2, the next step is to compute an upper threshold by finding the gene *h *without annotation *a *most similar to genes with the annotation. We consider this an upper bound threshold and will assign the annotation to gene *g *if its similarity to other genes with the annotation is higher than this threshold.(8)

**Figure 3:**
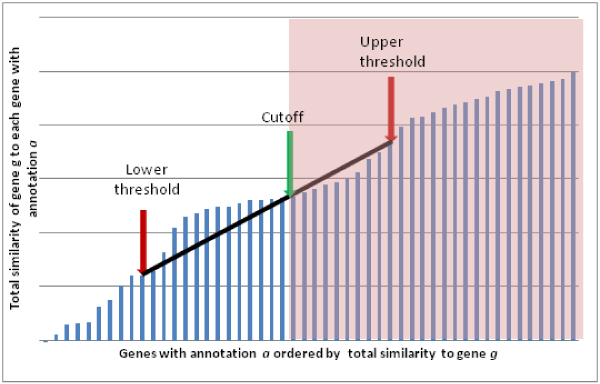
**Predicting transfer to annotations to genes**. When considering whether an annotation *a *should be assigned to gene *g*, the total similarity of each gene with annotation a to gene g is computed. If the total similarity is greater than the upper threshold, the annotation is assigned. If the total similarity is less than the lower threshold, the annotation is never assigned. For genes with a total similarity greater than the lower threshold but less than the upper threshold, linear interpolation is used to determine where the similarity falls relative to the two thresholds. If the interpolated similarity is above a predefined cutoff, the annotation is assigned. The pink area indicates similarity values for which the annotation will be transferred.

The third step is computation of the total similarity of gene *g *to all genes with annotation *a*. In the fourth step, a prediction decision is made based on the two thresholds and the total similarity as illustrated in Figure 3. If the total similarity is above the upper threshold, annotation *a *is assigned to the gene *g*. For total similarity values between the thresholds, we linearly interpolate between the thresholds and use a predefined cutoff to determine if the interpolated similarity is sufficiently high to assign the annotation *a *to gene *g*.

### Prediction metrics

Because the prediction algorithm given in Figure 1 only tests for a single gene and a single annotation at a time, it implicitly uses a jackknifing, or leave-one-out, approach for prediction [14]. In this approach, all of the genes except the one in question are used to make predictions about whether that gene should receive the annotation. A nice property of jackknifing is the small amount of bias it induces when considering the generalization of models [14]. Metrics assessing the quality of the predictions can be computed by comparing the annotations predicted for gene *g *when the gene is "left out" with the annotations already assigned to that gene.

As mentioned previously, the algorithm uses a cutoff to distinguish between positive and negative predictions. In our experiments, we use a series of cutoffs. For example, with a cutoff value of 0.6, we would test if the interpolated similarity (*interpolated_sim *in the pseudocode in Figure 1) for a particular gene and annotation is greater than 0.6. If it is, then the gene is predicted to have the annotation. In all cases, annotations with fewer than five genes known to have the annotation are disregarded. For comparison, we have predicted annotations using a combination of gene expression and textual phenotype data, using gene expression data alone, and using phenotype data alone. All results referring to predictions made using the graph constructed from the combination of the data sets and taking the sum of the similarities will be referred to as results from the "integrated sum data set," and those from the graph constructed from the combined data sets and taking the maximum of the similarities will be referred to as results from the "integrated max data set." Results referring to predictions from the gene expression graph will be referred to as results from the "gene expression data set," and those from the phenotype data will be referred to as results from the "phenotype data set."

The accuracy of the predictions is assessed using precision, recall and F-measure. Precision is the fraction of the annotation assignments (positive predictions) that are correct:(9)

where *tp *(true positives) is the number of correct positive predictions and *fp *(false positive) is the number of incorrect positive predictions [16]. Recall reflects the fraction of known annotations found by the algorithm:(10)

where *fp *(false negatives) are genes known to have an annotation but predicted as not having the annotation [16]. The precision and recall measures give complementary views of the effectiveness of a prediction algorithm and selecting a cutoff to increase one measure will typically decrease the other measure. The F-measure combines these views into a single metric and allows one to tradeoff precision and recall. In general, the F-measure can place more or less importance on precision as compared to recall [11]. For our experiments, the two were weighted equally:(11)

### Gene expression experimental data

All gene expression data for these experiments was downloaded from GEO [7] on November 5, 2008. Yeast expression data were used. All of the data was generated on the GPL1914 platform [15], which uses a spotted DNA/cDNA approach. The data are all normalized using the Rosetta Resolver approach [16]. Table 1 lists the GEO accessions and titles of all of the samples used.

**Table 1:** GEO Accessions

Sample	Sample Title
GSM112158	Yeast cell cycle-time point 0 min 2001-10-30_O.rfm Yeast W303 cells
GSM112159	Yeast cell cycle-time point 5 min 2001-11-09_0005.rfm Yeast W303 cells
GSM112160	Yeast cell cycle-time point 10 min 2001-11-09_0010.rfm Yeast W303 cells
GSM112161	Yeast cell cycle-time point 15 min 2001-11-09_0015.rfm Yeast W303 cells
GSM112162	Yeast cell cycle-time point 20 min 2001-11-09_0020.rfm Yeast W303 cells
GSM112163	Yeast cell cycle-time point 25 min 2001-11-09_0025.rfm Yeast W303 cells
GSM112164	Yeast cell cycle-time point 30 min 2001-11-09_0030.rfm Yeast W303 cells
GSM112165	Yeast cell cycle-time point 35 min 2001-11-09_0035.rfm Yeast W303 cells
GSM112166	Yeast cell cycle-time point 40 min 2001-11-09_0040.rfm Yeast W303 cells
GSM112167	Yeast cell cycle-time point 45 min 2001-11-09_0045.rfm Yeast W303 cells
GSM112168	Yeast cell cycle-time point 50 min 2001-11-09_0050.rfm Yeast W303 cells
GSM112169	Yeast cell cycle-time point 55 min 2001-11-09_0055.rfm Yeast W303 cells
GSM112170	Yeast cell cycle-time point 60 min 2001-11-09_0060.rfm Yeast W303 cells
GSM112171	Yeast cell cycle-time point 65 min 2001-11-21_0065.rfm Yeast W303 cells
GSM112172	Yeast cell cycle-time point 70 min 2001-11-21_0070.rfm Yeast W303 cells
GSM112173	Yeast cell cycle-time point 75 min 2001-11-28_0075.rfm Yeast W303 cells
GSM112174	Yeast cell cycle-time point 80 min 2001-11-28_0080.rfm Yeast W303 cells
GSM112175	Yeast cell cycle-time point 85 min 2001-11-29_0085.rfm Yeast W303 cells
GSM112176	Yeast cell cycle-time point 90 min 2001-11-29_0090.rfm Yeast W303 cells
GSM112177	Yeast cell cycle-time point 95 min 2001-11-29_0095.rfm Yeast W303 cells
GSM112178	Yeast cell cycle-time point 100 min 2001-11-29_0100.rfm Yeast W303 cells
GSM112179	Yeast cell cycle-time point 105 min 2001-12-06_0105.rfm Yeast W303 cells
GSM112180	Yeast cell cycle-time point 110 min 2001-11-29_0110.rfm Yeast W303 cells
GSM112181	Yeast cell cycle-time point 115 min 2001-11-29_0115.rfm Yeast W303 cells
GSM112182	Yeast cell cycle-time point 120 min 2001-11-29_0120.rfm Yeast W303 cells
GSM81064	Yeast cell cycle-time point 0 min 2001-05-03_0000.rfm
GSM81065	Yeast cell cycle-time point 10 min 2001-05-03_0010.rfm
GSM81066	Yeast cell cycle-time point 20 min 2001-05-03_0020.rfm
GSM81067	Yeast cell cycle-time point 30 min 2001-05-03_0030.rfm
GSM81068	Yeast cell cycle-time point 40 min 2001-04-11_0040.rfm
GSM81069	Yeast cell cycle-time point 50 min 2001-04-11_0050.rfm
GSM81070	Yeast cell cycle-time point 60 min 2001-04-11_0060.rfm
GSM81071	Yeast cell cycle-time point 70 min 2001-04-11_0070.rfm
GSM81072	Yeast cell cycle-time point 80 min 2001-04-11_0080.rfm
GSM81073	Yeast cell cycle-time point 90 min 2001-04-11_0090.rfm
GSM81074	Yeast cell cycle-time point 100 min 2001-04-11_0100.rfm
GSM81075	Yeast cell cycle-time point 110 min 2001-04-11_0110.rfm
GSM81076	Yeast cell cycle-time point 120 min 2001-04-11_0120.rfm

These data were downloaded from GEO on November 5, 2008. The data sets were generated by a variety of researchers in many different laboratories.

As previously mentioned, the identifiers for the gene expression data do not exactly correlate to single genes. Affymetrix provides a bridge which maps between expression identifiers and Entrez gene symbols [17]. Not all expression identifiers mapped to a gene symbol, and others mapped to more than one gene symbol. Only expression identifiers which mapped to a single gene symbol were retained. All other expression data was discarded. A total of 6251 expression identifiers were present in 39 expression runs. After mapping identifiers to Entrez gene symbols, 3169 entries remained. Therefore, each of the 3169 genes had an associated 39-dimensional array of expression values.

### Phenotype textual experimental data

The PhenomicDB http://www.phenomicdb.de/ incorporates data from many different data sources about a wide variety of organisms, including human, yeast, mouse, and many others [18]. The database provides a large number of searching options, including searching by Entrez gene symbols. For each of the gene symbols identified with gene expression values, PhenomicDB was consulted for phenotypes associated with that gene symbol in yeast. The data was downloaded on November 23, 2008.

In general, PhenomicDB contains multiple phenotypes for each gene symbol. Each phenotype is a textual description. To form a single document for each gene symbol, all of the phenotypes are simply concatenated. However, this plain text representation of knowledge does not easily lend itself to learning approaches.

The document associated with each symbol was transformed into a tf-idf array. The doc2mat utility from the CLUTO package [19] applies a stop word list and the Porter stemming algorithm to produce a term frequency description of each document [13]. A stop word list is used to remove common, uninformative words, such as articles and prepositions, from the documents. The stemming algorithm is used to remove prefixes and suffixes from words. The term frequency and inverse document frequency values for each term are multiplied to produce a tf-idf array for each document. A total of 6541 distinct terms were discovered after pruning and stop words were applied. Hence, each of the tf-idf arrays had 6541 dimensions. Each dimension in the array corresponds to one unique term. The value of each dimension is a fraction in which the numerator is the number of times the term corresponding to that dimension occurs in the document and the denominator is the total number of documents in which the term appears. Because the numerator cannot be less than 0 and the denominator cannot be less than 1, the resulting values are always nonnegative.

### Functional annotations

Our algorithm utilizes GO terms as labels. Fortunately, the file provided by Affymetrix which provides the mapping between expression identifiers and gene symbols also includes all GO terms associated with each gene symbol [17]. A total of 3,466 distinct GO annotations were identified in the Affymetrix file. A total of 39,680 annotation assignments were defined between the GO annotations and the 3,169 genes.

## Results and discussion

When considering the correctness of predictions, two different approaches were used. In the first case, only exact annotation matches are considered correct. For example, if predicting that gene *g *has annotation *a*, the prediction is considered a true positive only if *g *is labelled exactly with *a*. Otherwise, the prediction is a false positive. These are referred to as "exact" predictions. However, the Gene Ontology enforces the "true path rule" stating that "the pathway from a child term all the way up to its top-level parent(s) must always be true" [20]. This means that if annotation *a *is predicted for gene *g *and the gene has been previously assigned a GO term that is a child of *a*, the assignment of *a *to the gene *g *is also correct. Therefore, we use an alternate method of computing the number of correct predictions where, if predicting that gene *g *has annotation *a*, the prediction is considered a true positive if *g *is labelled exactly with *a *or with any child term of *a*. The second case is referred to as "generalized" predictions.

**Figure 4:**
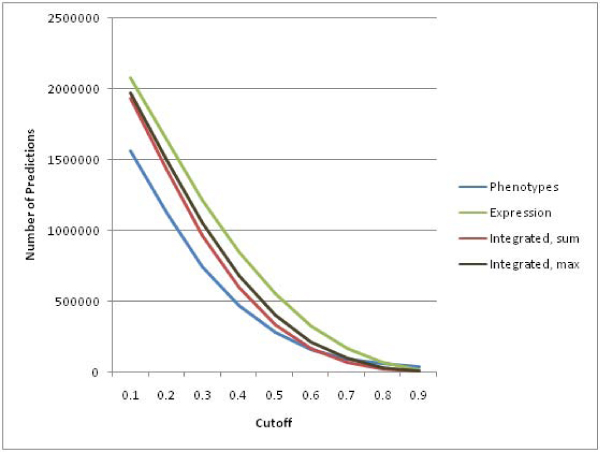
**Total Number of Positive Predictions**. As the *cutoff *used in the prediction algorithm is increased, all of the datasets make fewer positive predictions. That is, they predict that fewer genes should be annotated with a particular GO term. However, the number of predictions based on only the phenotype data is consistently far less than the number based on the expression data or the combined data set. This suggests that, in general, the phenotype data will not be as much aid in making novel predictions as the other data sets. There is no difference in the number of predictions assigned by either the generalized or exact approaches since those only differentiate between which predictions are considered correct.

**Figure 5:**
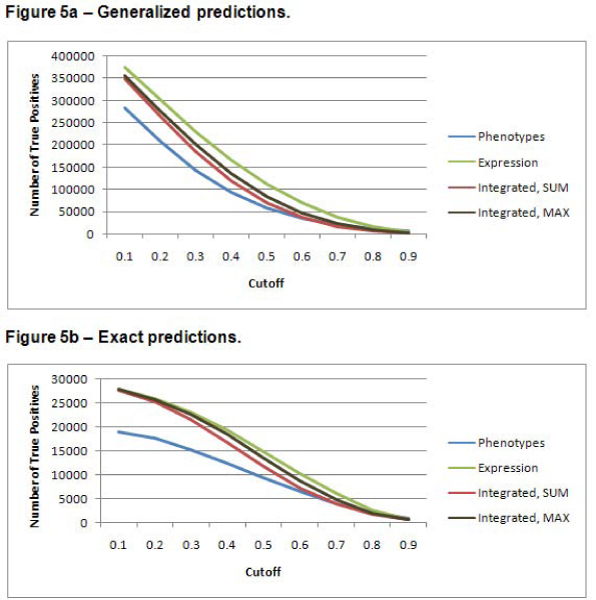
**Total number of correct positive predictions**. As the *cutoff *used in the prediction algorithm is increased, all of the datasets make fewer correct positive predictions. The integrated data set makes nearly as many correct predictions as the gene expression data set, and they both make many more predictions than the phenotype data set. This confirms that the phenotype data set is not as capable as the other data sets in predicting new annotations. The generalized predictions always result in more true positives. Figure 5a shows the results for the generalized predictions. Figure 5b shows the results for the exact predictions.

Figure 4 indicates the total number of GO annotations predicted for each of the three data sets. The MAX method for combining similarities results in more positive predictions of GO terms than the SUM method. Figure 5 shows the total number of correct GO terms assigned using both the exact and generalized scoring methods. As expected, the generalized scoring method gives a much higher number of correct assignments. Figure 4 and 5 indicate that, overall, the gene expression data set resulted in the most GO terms predicted and in the most and correct GO assignments. Both of the integrated approaches produced numbers of assignments and correct assignments that were only somewhat lower than those for the expression data set. The textual phenotype data set produced far fewer total assignments and correct assignments at all except the most stringent cutoff values. These results indicate that the gene expression and integrated approaches have the potential to discover many more new annotations than the phenotype data set.

### Precision, recall, and F-measure

**Figure 6:**
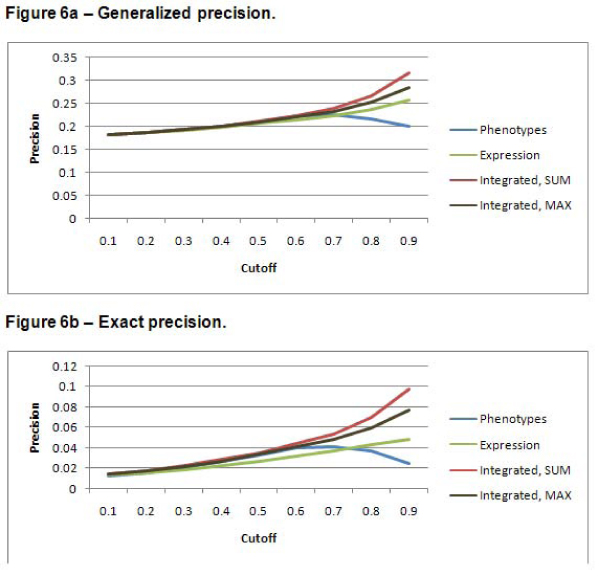
**Precision**. As the *cutoff *used in the prediction algorithm is increased, the precision of all of the data sets increases. Precision is defined as *(tp)/(tp + fp) *[16]. Combined with Figures 4 and 5, this indicates that, while fewer false positives are predicted as *cutoff *is increased, fewer true positives are also predicted. This is especially true in the case of the phenotype data set, which resulted in far fewer predictions than the other data sets. The integrated data set does outperform the other data sets. The generalized predictions result in a better precision than the exact predictions. Figure 6a shows the results for the generalized predictions. Figure 6b shows the results for the exact predictions.

**Figure 7:**
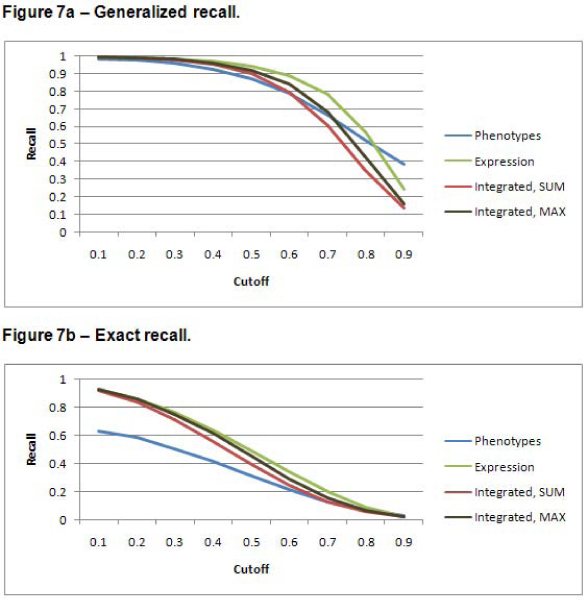
**Recall**. In contrast to recall, as the *cutoff *is increased, the recall decreases. Recall is defined as *(tp)/(tp + fn) *[16]. Since false negatives indicate negative predictions of known positive annotations, it is not surprising that the values would decrease as the *cutoff *is increased since that results in fewer predictions. The gene expression data set has the highest recall, but the integrated data set is only slightly lower. The generalized predictions have a better recall than the exact predictions. Figure 7a shows the results for the generalized predictions. Figure 7b shows the results for the exact predictions.

**Figure 8:**
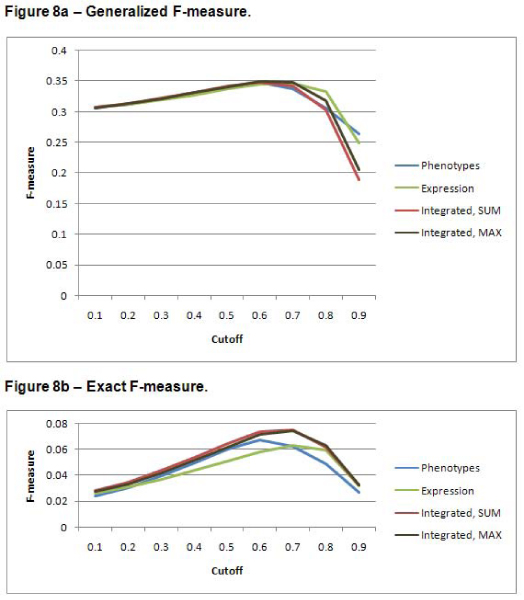
**F-measure**. The F-measure tends to favor *cutoffs *which are neither very high nor very low. F-measure is calculated as (2*precision*recall)/(precision + recall) [16]. Thus, the best F-measures strike a balance between precision and recall. The integrated data set using the max to combine the similarities results in the highest F-measure with a cutoff of 0.6. Figure 8a shows the results for the generalized predictions. Figure 8b shows the results for the exact predictions.

Figures 6, 7, and 8 show the precision, recall, and F-measure values respectively, for annotation predictions using each data source for a variety of cutoffs for the two scoring methods. The integrated approach results in improved precision over predictions based on either data set alone – especially for high cutoff values. Recall is higher for the expression dataset. These prediction results illustrate the precision/recall tradeoff problem. Because of the large number of negative samples, simply predicting that a gene should never have an annotation results in a very high precision; of course, such a scheme defies the point of developing an algorithm to predict functional predictions. The F-measure attempts to combine precision and recall into a single metric. The highest F-measure is obtained with a cutoff of 0.6. Note that this cutoff does not yield the highest precision or the highest recall. Because the precision values for all data sources are quite low, it might be preferable to use an F-measure metric that gives a higher weight to precision.

The results show that, as expected, the generalized scoring method yields higher precision, recall and F-measure values than the exact method. One can argue that the exact scoring method is unnecessarily strict and somewhat arbitrary because it requires the automated method to learn GO terms at exactly the same level as those assigned by expert annotators. In general, automated procedures tend to assign GO terms at higher levels than can be obtained by expert biocurators reading the literature. The "true path rule" of the Gene Ontology guarantees that the annotations scored as correct by the generalized scoring method are truly correct. The weakness of this scoring method is that more general terms are less informative than more specific terms. Although the precision values obtained using generalized scoring are substantially higher than those obtained with exact scoring, precision is still quite low. It should be noted that some of the GO term assignments scored as incorrect, may indeed be correct. Although yeast is one of the best annotated model organisms, annotation of yeast gene products is not complete and new annotations are constantly being added. In some cases the automated algorithm may have "learned" a more specific term than is currently assigned. Another factor contributing to the low precision is the type of gene expression data used. Because all of the experiments concern cell cycle, many of the genes do not have informative expression profiles. Including other types of gene expression data could help alleviate this problem and increase precision. The higher precision scored obtained by the integrated approach indicates that this approach allows one to take advantage of the large number of assignments that can be made based on gene expression data while at the same time gaining the precision afforded by the phenotype data.

### Prediction depths

**Figure 9:**
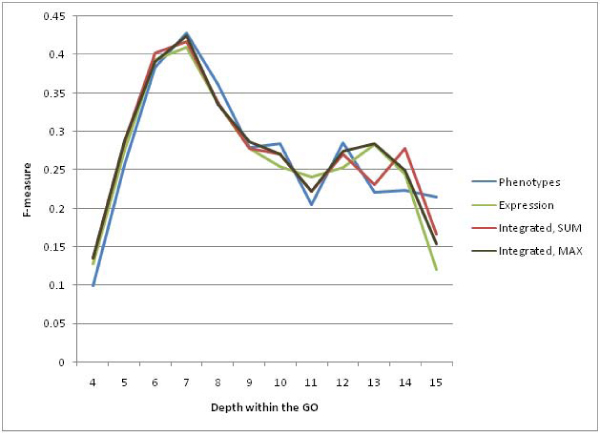
**F-measures at different depths within the GO**. Because of the more specific information available in the phenotype data set, it results in more accurate predictions at deeper levels in the GO. This figure shows that for predictions at levels between 7 and 12 in the GO, the phenotype data almost always has a higher F-measure. However, by combining the phenotype data with the expression data, the many more predictions (see Figures 4 and 5) made by the integrated data set do fare better than those of the gene expression data set alone. These were only evaluated using the generalized predictions. A cutoff of 0.6 was used in all cases.

Because of the differences in the nature of the textual phenotype data and the gene expression data, their performance at predicting annotations at different depths in the GO was also investigated. Terms deeper in the GO are more specific and thus more informative. Figure 9 demonstrates how the F-measure at different depths in the GO hierarchy varies for each data source. Although the phenotypic data provides the highest F-measure deeper in the hierarchy, this type of data resulted in far fewer predicted annotations than the gene expression data. The integrated approach improves the F-measure values deep in the hierarchy over what is obtained by the gene expression data alone.

In summary, the integrated approach results in nearly as many annotation predictions as the gene expression data, as indicated in Figure 3, but still maintains much of the precision of the phenotype data set, as shown in Figure 5.

### Biologically relevant results

The integrated methods do produce biologically relevant predictions which are not made by the individual data sets. For example, the *Saccaromyces *Genome Database http://yeastgenome.org/ indicates that the gene PDR11 is a "multidrug transporter involved in multiple drug resistance." While it is annotated with GO:0015918 (sterol transport) and GO:0042626 (ATPase activity, coupled to transmembrane movement of substances), it is not explicitly annotated with any functions related to multidrug transport. The MAX integrated data set predicts that it should be annotated with "multidrug transport," GO: 0006855. The gene expression data set alone is not able to make this prediction. As another example, the MAX integrated data set predicts that SPT21 should be annotated with GO: 0006348 "chromatin silencing at telomere." The *Saccaromyces *Genome Database description of SPT21 states that the gene is involved in telomere maintenance; however, it is not annotated with any GO molecular functions. This prediction is not made when using only the phenotype data set. These examples demonstrate that not only can the prediction algorithm make novel predictions consistent with biological knowledge, but also that integrating the data types can result in predicted annotations that either individual data set alone would fail to identify.

## Conclusion

This paper presents an algorithm that incorporates both gene expression data and textual phenotype data to predict the function of genes. This graph-based approach generates a complete graph weighted with gene-gene similarities. It then makes predictions based on the weights connecting the nodes. The results indicate that integrating the gene expression with the textual phenotypes produces more precise annotations than predictions based upon either type of data alone.

The integrated approach outperformed the gene expression-only graph in the precision metric; it also tended to outperform the textual phenotype graph in the recall metric. Furthermore, the integrated similarity graph produced many more correct annotation assignments than the phenotype graph alone. We believe that this integrated approach can augment the usefulness of standard gene expression data by facilitating annotation predictions with increased precision and an increased F-measure deeper within the GO hierarchy.

Future work could focus on development of better methods to integrate the data sets. For example, rather than equally weighting the gene expression and textual data, methods could be developed for assigning different weights to different data types when determining the edge weights. A less naïve integration method could be used to map the correlation and cosine values to more meaningful numbers, such as p-values.

## Competing interests

The authors declare that they have no competing interests.

## Authors' contributions

BMM implemented the algorithm and carried out the experiments. ADP contributed to the algorithm and sources for experimental data. SMB helped draft the document and design experiments. All authors read and approved the final manuscript.
